# Proposing the lymphatic target volume for elective radiation therapy for pancreatic cancer: a pooled analysis of clinical evidence

**DOI:** 10.1186/1748-717X-5-28

**Published:** 2010-04-15

**Authors:** Wenjie Sun, Cheng N Leong, Zhen Zhang, Jiade J Lu

**Affiliations:** 1 Department of Radiation Oncology, Fudan University Shanghai Cancer Center Department of Oncology, Shanghai Medical College, Fudan University, Shanghai 200032, China; 2 Department of Radiation Oncology, National University Cancer Institute, National University Health System, National University of Singapore, Singapore 119074, Republic of Singapore

## Abstract

**Background:**

Radiation therapy is an important cancer treatment modality in both adjuvant and definitive setting, however, the use of radiation therapy for elective treatment of regional lymph nodes is controversial for pancreatic cancer. No consensus on proper selection and delineation of subclinical lymph nodal areas in adjuvant or definitive radiation therapy has been suggested either conclusively or proposed for further investigation. This analysis aims to study the pattern of lymph node metastasis through a pooled analysis of published results after radical tumor and lymph nodal resection with histological study in pancreatic cancer.

**Methods:**

Literature search using electronic databases including MEDLINE, EMBASE, and CANCERLIT from January 1970 to June 2009 was performed, supplemented by review of references. Eighteen original researches and a total of 5954 pancreatic cancer patients underwent radical surgical resection were included in this analysis. The probability of metastasis in regional lymph nodal stations (using Japan Pancreas Society [JPS] Classification) was calculated and analyzed based on the location and other characteristics of the primary disease.

**Results:**

Commonly involved nodal regions in patients with pancreatic head tumor include lymph nodes around the common hepatic artery (Group 8, 9.79%), posterior pancreaticoduodenal lymph nodes (Group 13, 32.31%), lymph nodes around the superior mesenteric artery (Group 14, 15.85%), paraaortic lymph nodes (Group 16, 10.92%), and anterior pancreaticoduodenal lymph nodes (Group 17, 19.78%); The probability of metastasis in other lymph nodal regions were <9%.

Commonly involved nodal regions in patients with pancreatic body/tail tumor include lymph nodes around the common hepatic artery (Group 8, 15.07%), lymph nodes around the celiac trunk (Group 9, 9.59%), lymph nodes along the splenic artery (Group 11, 35.62%), lymph nodes around the superior mesenteric artery (Group 14, 9.59%), paraaortic lymph nodes (Group 16, 16.44%), and inferior body lymph nodes (Group 18, 24.66%). The probability of metastasis in other lymph nodal regions were <9%.

**Conclusions:**

Pancreatic cancer has a high propensity of regional lymphatic metastases; however, clear patterns including the site and probability of metastasis can be identified and used as a guide of treatment in patients with resectable pancreatic cancer. Further clinical investigation is needed to study the efficacy of elective treatment to CTV defined based on these patterns using high-dose conformal or intensity-modulated radiation therapy.

## Introduction

Pancreatic cancer is a highly malignant neoplasm of GI system, and radical surgery is its only curative treatment option [1]. Unfortunately, the probability of locoregional recurrence approaches 80% after complete resection, and long-term survival is less than 25% even for patients treated for early stage disease [2-4]. Adjuvant treatment is an integral part of definitive treatment of resectable pancreatic carcinoma; however, the optimal therapeutic modalities in adjuvant setting remain a focus of debate.

Radiation therapy is commonly used in adjuvant treatment for pancreatic cancer after radical surgery in the United States. The effect of radiation with concurrent 5-FU based chemotherapy has been suggested in a number of randomized clinical trials [5-7]. In addition, concurrent chemoradiation therapy has been the mainstay treatment for nonmetastatic and inoperable pancreatic cancer [8,9].

Radiation fields utilized in these trials encompassed not only subclinical nodal regions but also adjacent normal tissues. Despite such generous coverage, however, locoregional control remains a major mode of recurrence. The underlying reason for such suboptimal outcome is probably due to, at least in part, insufficient radiation dose (i.e., 45-50 Gy in conventional fractionation) to the surgical bed and draining lymph node regions limited by the organs at risk (OARs) adjacent to the pancreas and lymph nodal regions such as liver, small intestine, stomach, spinal cord, and kidneys.

The prevailing utilization of intensity-modulated radiation therapy (IMRT) in cancer treatment including upper GI malignancies enabled dose differentiation between target volumes and adjacent normal tissues and organs thereby improved therapeutic ratio. Results from recently published dosimetry studies have suggested the advantage of IMRT in the treatment of tumors of upper abdomen including pancreatic, gastric, and billiary cancers as compared to 3-dimentional conformal radiation therapy (3D-CRT) [10-13]. Proper defining of high-risk regions especially the lymph nodal regions (i.e., CTV-N) forms an imperative basis for dose escalation using IMRT. However, selection and delineation of nodal regions in both adjuvant IMRT after pancreaticoduodenectomy and in definitive setting have never been addressed.

The aim of this analysis is to address the selection of high-risk subclinical lymph nodal regions in conformal radiation therapy for resectable pancreatic cancer, by reviewing and summarizing the probability of lymph node metastases in resectable pancreatic cancer patients treated with radical surgery with lymph node dissection and pathological investigation of the resected regional nodes.

## Methods

An exhaustive search and review of original articles analyzing lymph nodal positivity rate of pancreatic cancer was performed by searching MEDLINE, EMBASE, and CANCERLIT from January 1970 to June 2009. The search strategy used the following key words in various combinations: "pancreatic cancer", "pancreatic carcinoma", "lymph node", and "surgery". Based on the titles of the articles, studies not describing the pattern of lymph nodal metastasis were excluded, and the entire article of those retained and published in English were read and screened. Studies were eligible if lymph node positivity rates in pancreatic cancer were reported. We also supplemented correlative articles by reading the references of reviews.

**Figure 1:**
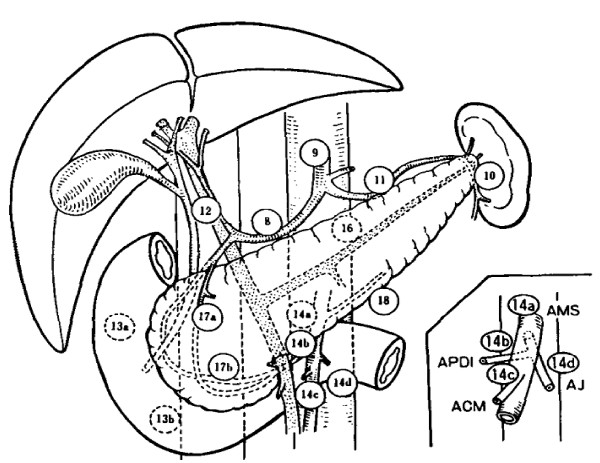
**JPS Classification of the regional lymph nodes of the pancreas**. (Adapted from Nagakawa T, Kobayashi H, Ueno K, Ohta T, Kayahara M, Mori K, Nakano T, Takeda T, Konishi I, Miyazaki I: The pattern of lymph node involvement in carcinoma of the head of the pancreas. A histologic study of the surgical findings in patients undergoing extensive nodal dissections. Int J Pancreatol. 1993,13:15-22 [19]. Used with permission from Springer Science+Business.) Insert: Subdivision of Group 14: (AMS) superior mesenteric artery; (AJ) jejunal artery; (APDI) inferior pancreaticoduodenal artery; (ACM) medial colic artery; For other abbreviations of the nodal groups refer to Table 2.

All lymph nodes were classified according to the General Rules for Cancer of the Pancreas published by the Japan Pancreas Society (JPS) (Figure 1) [14]. Articles in which dissected lymph nodes could not be classified according to the rule of JPS were excluded. There was no restriction criterion on the number of patients enrolled in the study.

The rate of disease involvement of all lymph nodal regions (according to JPS Classification) was the primary outcome. Relationship between lymph nodal metastasis and tumor characteristics (T classification, lymphatic vessel invasion, tumor location, tumor size and tumor differentiation) was also evaluated.

The accuracy of data from individual publication including the conversion to JPS lymph node classification was examined by two participants of this analysis. Pooled analyses of the rates of metastasis to lymph nodal regions were calculated and reported. Statistical correlation between metastasis to lymph node areas and tumor characteristics was performed using Fisher's exact test.

## Results

### Characteristics of Included Studies

**Figure 2:**
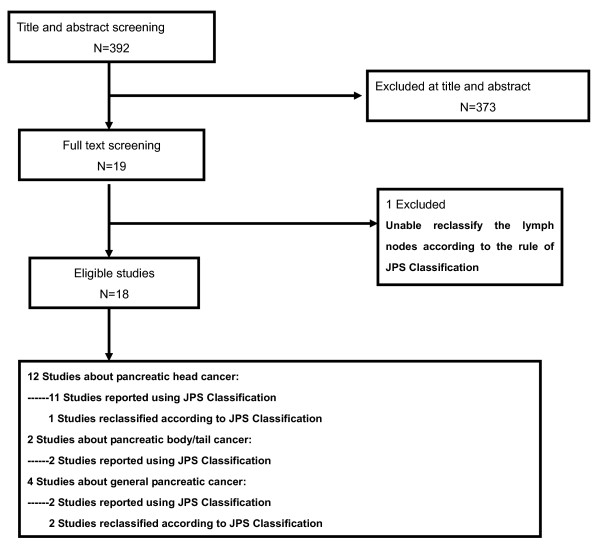
**Flowchart of studies to final number of eligible studies**.

The initial search resulted in 392 citations. The title and abstract of each retrieved publication were reviewed to confirm that the article reported on the incidence of lymph nodal positivity in patients with pancreatic cancer. In the event that this approach was not informative, the full article was retrieved and reviewed in detail. This process resulted in excluding 373 studies and 19 studies were selected. Of these 19 studies, one study [15] was further excluded from this analysis because we could not classify the lymph nodes of this study according to the rule of JPS (Figure 2).

In 18 eligible studies [1,16-32], 12 (66.7%) studies described lymph nodal metastatic rates of pancreatic head cancer; cancer of body/tail of pancreas in only 2 (11.1%) studies and general pancreatic cancer (including all locations of pancreas) in 4 (22.2%) studies (Table 1). In analyzing the metastatic pattern of lymph nodes, we divided the data of one study about general pancreatic cancer (Study No.3) [17] into two parts (the data about pancreatic head cancer and those about pancreatic body/tail cancer), then integrated these two parts into the statistical analysis of pancreatic head and body/tail cancer respectively.

**Table 1:** Classification of regional lymph nodes of the pancreas

No. of Study	Studies	Year	Location of Tumors	No. of Patients	LN Positivity
1	Brunner TB [16]	1978-1997	Head	178	NA

2	Matsuno S [1]	1981-2002	Head	4913	NA

3	Kayahara M [17]	1974-1996	General*	99	78%

4	Sierzega M [18]	1980-2002	Head	96	66.70%

5^#^	Nagakawa T [19]	1973-1989	Head	42	78.60%

6	Nagakawa T [20]	1973-1989	Head	42	78.60%

7	Capussotti L [21]	1988-1998	Head	100	59%

8	Cubilla AL [22]	1974-1976	General*	22	86.40%

9	Nakao A [23]	1981-1995	Body/tail	30	46.70%

10	Nagai H [24]	1980-1983	General*	8	75%

11	Kayahara M [25]	1980-1994	Body/tail	20	80%

12	Kayahara M [26]	N/A	Head	44	70.50%

13	Yoshida T [27]	1995-1999	Head	34	NA

14	Sakai M [28]	1981-2002	Head	178	66%

15	Gerdes B [29]	1995-2002	Head	50	64%

16	Ishikawa O [30]	1981-1994	Head	81	73%

17	Kayahara M [31]	1973-1991	Head	49	76%

18	Kocher HM [32]	N/A	General*	10	80%

^#^The patients of Study No.5 are same as those of Study No.6.

N/A = not applicable; * "General" indicates the location of tumor in that study include either head or body/tail.

The 18 studies, which met the inclusion criteria, reported on 5954 pancreatic cancer patients who had undergone radical lymph nodes dissections (Table 1). 17 studies were prospective, and the remaining one was a statistic summary of 20 years' registry results on JPS website [1]. The median number of pancreatic cancer patients enrolled per study was 49.5 (range 8-4913 patients). In studies that provided baseline demographic information on pancreatic cancer patients, 414 were men and 242 were women [16-23]. The mean age was reported in 5 studies and ranged between 61 years and 74.8 years [17,23-25,28], and the reported median age ranged from 59 years to 65 years in 3 studies [16,18,29].

Twelve of 18 (66.7%) studies commented on the histopathologic examination of lymph nodes, which consisted of routine hematoxylin and eosin staining, with additional sections evaluated by elastica van Gieson staining in 4 (33.3%) studies [17,25,26,32] and by elastic-Masson staining in 1 (8.3%) study [24]. Furthermore, 16 of 18 (88.9%) studies described the histopathologic type of pancreatic cancer. Of these, almost all enrolled patients' histopathologic types were ductal adenocarcinomas except for two patients. One patient's histopathological type was undifferentiated carcinoma and another was adenosquamous carcinoma.

### Regional lymph node metastasis pattern based on different locations of tumors

**Figure 3:**
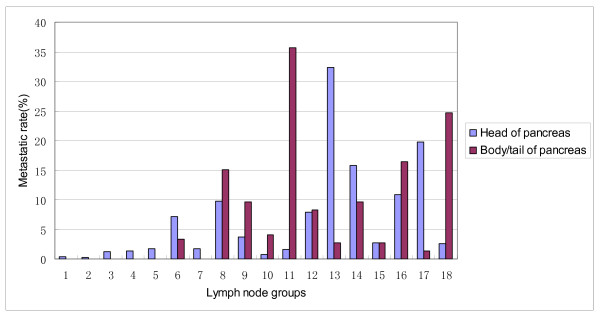
**Frequency of lymph nodal metastasis of pancreatic cancer**.

The probability of metastasis in regional lymph nodal stations was calculated and analyzed by Japan Pancreas Society (JPS) Classification [14]. For all 5954 patients with pancreatic cancer (including head and body/tail of pancreas), commonly involved regional lymph nodal stations were lymph nodes around the common hepatic artery (Group8, 9.84%), posterior pancreaticoduodenal lymph nodes (Group13, 32.1%), lymph nodes around the superior mesenteric artery (Group14, 15.76%), paraaortic lymph nodes (Group16, 11.04%), anterior pancreaticoduodenal lymph nodes (Group17, 19.65%). Nodal sites with a frequency of metastasis <5% included right cardial lymph nodes (Group1, 0.39%), left cardial lymph nodes (Group2, 0.28%), lymph nodes along the lesser curvature of the stomach (Group3, 1.2%), lymph nodes along the greater curvature of the stomach (Group4, 1.37%), suprapyloric lymph nodes (Group5, 1.68%), lymph nodes around the left gastric artery (Group7, 1.73%), lymph nodes around the celiac trunk (Group9, 3.75%), lymph nodes at the hilus of the spleen (Group10, 0.84%), lymph nodes along the splenic artery (Group11, 1.93%), lymph nodes along the middle colic artery (Group15, 2.7%), inferior body lymph nodes (Group18, 3.04%) (Table 2, Figure 3).

**Table 2:** Metastatic rates of all groups of lymph nodes (According to JPS Classification)

No. Group No. Study	1 RC	2 LC	3 LCS	4 GCS	5 SP	6 IP	7 LGA	8 CHA	9 CT	10 HS	11 SA	12 HDL	13 PPD	14 SMA	15 MCA	16 PA	17 APD	18 IB
Pancreatic Head Cancer																		

1									8/175	2/175		31/175	65/175	17/175		39/175	41/175	

2	12/2974	8/2768	48/3796	57/3928	72/3973	298/4167	70/3697	728/7453	130/3697	23/2759	121/8260	921/12400	2588/8503	1182/7962	97/3364	501/5134	1524/8148	84/3266

3*								5/76	3/76	0/76	1/76	11/76	75/76	26/76	0/76	14/76	35/76	0/76

4	0/96	0/96	0/96	0/96	0/96	0/96	0/96	12/96	6/96	1/96	3/96	14/96		11/96	0/96	10/96		0/96

5(6)#			0/42	0/42	0/42	1/42	0/42	6/42	2/42	0/42	2/42	9/42	29/42	16/42	0/42	7/42	17/42	5/20

7					0/100	12/100		6/100	9/100			3/100		22/100	3/100			

12					0/44	1/44	0/44	6/44	2/44	0/44	2/44	6/44	28/44	15/44	0/44	7/44	14/44	4/44

13																9/34		

14						21/178	0/178	17/178	2/178	0/178	14/178	33/178	83/178	50/178	2/178	34/178	51/178	3/178

15			0/50	0/50														

16							2/81	9/81	2/81	1/81		12/81	40/81	38/81	5/81	15/81	30/81	

17								6/49	2/49			6/49	48/49	18/49		9/49	27/49	

Total (head)	12/3070	8/2864	48/3984	57/4116	72/4255	333/4627	72/4138	795/8119	166/4538	27/3451	143/8696	1046/13241	2956/9148	1395/8803	107/3981	645/5909	1739/8793	96/3680

^1^Total rate (head) %	0.39	0.28	1.2	1.38	1.69	7.2	1.74	9.79	3.66	0.78	1.64	7.9	32.31	15.85	2.69	10.92	19.78	2.61

Pancreatic Body/tail Cancer																		

3*								5/23	1/23	1/23	11/23	3/23	1/23	1/23	1/23	4/23	0/23	9/23

9	0/30	0/30	1/30	0/30	0/30	1/30	0/30	1/30	4/30	1/30	5/30	0/30	0/30	4/30	0/30	4/30	1/30	2/30

11								5/20	2/20	1/20	10/20	3/20	1/20	2/20	1/20	4/20	0/20	7/20

Total (body/tail)	0/30	0/30	0/30	0/30	0/30	1/30	0/30	11/73	7/73	3/73	26/73	6/73	2/73	7/73	2/73	12/73	1/73	18/73

^1^Total rate (Body/tail)%	0	0	0	0	0	3.33	0	15.07	9.59	4.11	35.62	8.22	2.74	9.59	2.74	16.44	1.37	24.66

General Pancreatic Cancer																		

8										0/22		0/22	10/22	1/22	1/22		2/22	

10										0/8		0/8	4/8			4/8	2/8	

18						1/10						1/10	1/10	1/10			6/10	

Total (general)						1/10				0/30		1/40	15/40	2/32	1/22	4/8	10/40	

^1^Total rate (general)%						10				0		2.5	37.5	6.25	4.55	50	25	

Total patients																		

Total	12/3100	8/2894	48/4014	57/4146	72/4285	335/4667	72/4168	806/8192	173/4611	30/3554	169/8769	1053/13354	2973/9261	1404/8908	110/4076	661/5990	1750/8906	114/3753

^2^Total rate%	0.39	0.28	1.2	1.37	1.68	7.18	1.73	9.84	3.75	0.84	1.93	7.89	32.1	15.76	2.7	11.04	19.65	3.04

Abbreviations:

Group1: right cardial lymph nodes (RC)

Group2: left cardial lymph nodes (LC)

Group3: lymph nodes along the lesser curvature of the stomach (LCS)

Group4: lymph nodes along the greater curvature of the stomach (GCS)

Group5: suprapyloric lymph nodes (SP)

Group6: infrapyloric lymph nodes (IP)

Group7: lymph nodes around the left gastric artery (LGA)

Group8: lymph nodes around the common hepatic artery (CHA)

Group9: lymph nodes around the celiac trunk (CT)

Group10: lymph nodes at the hilus of the spleen (HS)

Group11: lymph nodes along the splenic artery (SA)

Group12: lymph nodes of the hepatoduodenal ligament (HDL)

Group13: posterior pancreaticoduodenal lymph nodes (PPD)

Group14: lymph nodes around the superior mesenteric artery (SMA)

Group15: lymph nodes along the middle colic artery (MCA)

Group16: paraaortic lymph nodes (PA)

Group17: anterior pancreaticoduodenal lymph nodes (APD)

Group18: inferior body lymph nodes (IB)

^1^Total rate (head or body/tail or general) = (patient number with positive nodes in all studies mentioning such group of lymph node)/(patient number of all studies mentioning such group of lymph node) (for head or body/tail or general)

^2^Total rate = (patient number with positive nodes in all studies mentioning such group of lymph node)/(patient number of all studies mentioning such group of lymph node) (for all pancreatic cancers)

*The data of Study No.3 was divided into 2 parts according to the location of the tumor.

^#^Study No.5 had the same patients with Study No.6.

In 13 studies on pancreatic head cancer (including a part of data in Study No.3), metastatic rates of regional lymph nodes of 5838 patients were analyzed. Commonly involved nodal regions in patients with pancreatic head tumor included the posterior pancreaticoduodenal lymph nodes (Group 13, 32.31%), anterior pancreaticoduodenal lymph nodes (Group 17, 19.78%), lymph nodes around the superior mesenteric artery (Group 14, 15.85%), paraaortic lymph nodes (Group 16, 10.92%) and lymph nodes around the common hepatic artery (Group 8, 9.79%). The probability of lymph nodal metastasis <5% included right cardial lymph nodes (Group 1, 0.39%), left cardial lymph nodes (Group 2, 0.28%), lymph nodes along the lesser curvature of the stomach (Group 3, 1.2%), lymph nodes along the greater curvature of the stomach (Group 4, 1.38%), suprapyloric lymph nodes (Group 5, 1.69%), lymph nodes around the left gastric artery (Group 7, 1.74%), lymph nodes around the celiac trunk (Group 9, 3.66%), lymph nodes at the hilus of the spleen (Group 10, 0.78%), lymph nodes along the splenic artery (Group 11, 1.64%), lymph nodes along the middle colic artery (Group 15, 2.69%) and inferior body lymph nodes (Group 18, 2.61%) (Table 2, Figure 3).

There were 3 studies including 73 patients with cancer of body/tail of pancreas which were analyzed (including a part of data in Study No.3). Commonly involved nodal regions in patients with pancreatic body/tail tumor included lymph nodes around the common hepatic artery (Group 8, 15.07%), lymph nodes around the celiac trunk (Group 9, 9.59%), lymph nodes along the splenic artery (Group 11, 35.62%), lymph nodes around the superior mesenteric artery (Group 14, 9.59%), paraaortic lymph nodes (Group 16, 16.44%), inferior body lymph nodes (Group 18, 24.66%). The probability of lymph nodal metastasis <5% included Groups 1-5 and Group 7 (0%), Group 6(3.33%), Group 10(4.11%), Groups 13,15 (2.74%) and Group 17 (1.37%) (Table 2, Figure 3).

### Metastatic rates of subgroups of lymph nodes

We also analyzed the metastatic rates of several subgroups of lymph nodes based on tumor locations. The metastatic rates of subgroups of lymph nodes for patients with pancreatic head cancer were listed in Table 3 (not including paraaortic lymph nodes). For patients with pancreatic head cancer, commonly involved lymph nodal subgroups were 8a, 12b, 13a, 13b, 14a, 14b, 14d, 17a and 17b. However, tumor rarely spread to proximal and distal splenic lymph nodes (11p, 11d), lymph nodes around the proper hepatic artery (12a) which had <5% metastatic rates. The distribution of lymph nodal subgroups (not including paraaortic lymph nodes) for patients with pancreatic body/tail tumor was not described in any study.

**Table 3:** Metastatic rates of subgroups of lymph nodes of pancreatic head cancer (paraaortic lymph nodes not included)

No. Study	8a	8p	11p	11d	12a	12p	12b	13a	13b	14a	14b	14c	14d	17a	17b
3								40/76	35/76	13/76	16/76	5/76	9/76	22/76	13/76

6								22/42	19/42	8/42	11/42	3/42	6/42	13/42	9/42

2	523/3695	205/3030	121/3229	0/4910	180/3887	257/3808	484/3784	1490/2830	1098/3085					764/3282	760/3342

12								22/44	18/44	8/44	9/44	3/44	6/44	11/44	7/44

17								25/49	23/49	10/49	11/49	3/49	7/49	16/49	11/49

Metastatic rate%	14.2	6.8	3.7	0	4.6	6.7	12.8	52.6	36.2	18.5	22.3	6.6	13.3	23.6	22.5

Abbreviations:

8a = lymph nodes of the anterior-superior region (group 8)

8p = lymph nodes of the posterior region (group 8)

11p = proximal splenic lymph nodes (group 11)

11d = distal splenic lymph nodes (group 11)

12a = lymph nodes around the proper hepatic artery (group 12)

12p = lymph nodes around the portal vein (group 12)

12b = lymph nodes around the bile duct (group 12)

13a = lymph nodes above the papilla of Vater (group 13)

13b = lymph nodes below the papilla of Vater (group 13)

14a = lymph nodes at the root of the superior mesenteric artery (group 14)

14b = lymph nodes at the root of the inferior pancreatoduodenal artery (group 14)

14c = lymph nodes at the root of the medial colic artery (group 14)

14d = lymph nodes at the root of the intestinal arteries (group 14)

17a = lymph nodes above the papilla of Vater (group 17)

17b = lymph nodes below the papilla of Vater (group 17)

In addition, the distribution of paraaortic lymph nodes based on the locations of tumors was analyzed [17,20,26]. Similar lymph nodal distributions for disease from pancreatic head cancer and body/tail cancer were found. Wherever the primary tumors were situated, the majority of the positive lymph nodes were located in the areas between the celiac artery and the inferior mesenteric artery (metastatic rate of pancreatic head cancer: 17.3%; metastatic rate of pancreatic body/tail cancer: 17.4%), while other areas including those superior to the celiac artery or inferior to the inferior mesenteric artery had <2% in metastatic rates. In the areas between the celiac artery and the inferior mesenteric artery, the positive lymph nodes were mainly located anterior to the abdominal aorta (Area pre-aor) and the area between the abdominal aorta and the inferior vena cava (Area inter) (metastatic rate of pancreatic head cancer: Area pre-aor 8.6%, Area inter 11.7%; metastatic rate of pancreatic body/tail cancer: Area pre-aor 13%, Area inter 13%), while other areas including those posterior and lateral to the aorta and the vena cava and those anterior to the vena cava had <4% in metastatic rates.

### Correlation between metastasis to lymph nodes and tumor characteristics

We analyzed the correlation between the metastatic rates of all groups of lymph nodes and tumor characteristics (T stage, tumor differentiation, lymphatic vessel invasion and tumor size). There were three studies describing the distribution of lymph nodes based on tumor characteristics, 2 about pancreatic head cancer and 1 about pancreatic body/tail tumor. Two studies about pancreatic head cancer analyzed 6 groups of lymph nodes (according to JPS Classification), including lymph nodes around the celiac trunk (group 9), lymph nodes of the hepatoduodenal ligament (group 12), posterior pancreaticoduodenal lymph nodes (group 13), lymph nodes around the superior mesenteric artery (group 14), paraaortic lymph nodes (group 16), anterior pancreaticoduodenal lymph nodes (group 17). Altogether, there were only a few sites where frequency of spread was correlated with tumor characteristics, including group 12 and group 13 (these two groups correlated with lymphatic vessel invasion). One study about pancreatic body/tail cancer analyzed 8 groups of lymph nodes, including lymph nodes around the common hepatic artery (group 8), lymph nodes around the celiac trunk (group9), lymph nodes along the splenic artery (group 11), lymph nodes of the hepatoduodenal ligament (group 12), posterior pancreaticoduodenal lymph nodes (group 13), lymph nodes around the superior mesenteric artery (group 14), paraaortic lymph nodes (group 16), inferior body lymph nodes (group 18). Eventually, there was no significant correlation between distribution of lymph nodes and tumor characteristics.

## Discussion

Pancreatic cancer is a highly aggressive GI malignancy. The outcome of patients with pancreatic cancer, even after complete surgical resection for early stage diseases is usually dismal, and locoregional recurrence is a major mode of treatment failure in both resected and unresectable cases. Radiation therapy is a major cancer treatment modality in both adjuvant and definitive settings; however, its use in pancreatic cancer, either definitively or adjuvantly, has been a focus of debate [33]. The suboptimal outcome after radiation therapy is due to, at least in part, insufficient dose to both gross and subclinical regions [33,34].

Irradiation to a large abdominal volume using conventional radiation to a high dose usually induces severe treatment-related side effects and complications. The advances in radiation therapy technology especially the use of IMRT have made precision targeting with high dose radiation therapy possible in the treatment of upper abdominal disease [11,12,35]. However, optimal strategy of selection and delineation of the subclinical regional disease in clinical target volume (CTV) in the treatment of pancreatic cancer has not been addressed. With more effective chemotherapy for systemic treatment of pancreatic cancer, effective local therapy to both tumor/surgical bed and the subclinical regional lymph node regions may become one of the deterministic factors for disease control in the treatment of non-metastatic pancreatic cancer. As the subclinical involvement of lymph nodes cannot be reliably discovered by image studies including CT, MRI, and/or PET/CT [36-38], proper selection and delineation of CTV should be accounted for the major challenge for radiation oncologists in the treatment of this disease. However, no evidence-based recommendations for target volume definition especially CTV have been provided for conformal radiation therapy for pancreatic cancer.

The current study analyzed all available clinical evidence on the pattern of lymph node metastases based on pathological examination after definitive surgery, and concluded that the pattern, namely the probability and sites of lymph node metastasis from tumors originated from the head or the body/tail of the pancreas can be followed. In patients with pancreatic head cancer, the most commonly involved lymph node regions include lymph nodes around the common hepatic artery, posterior pancreaticoduodenal lymph nodes, lymph nodes around the superior mesenteric artery, paraaortic lymph nodes and anterior pancreaticoduodenal lymph nodes. These nodal regions should be considered as the high-risk regions and encompassed in CTV. Some of the above-mentioned nodal groups can be further differentiated anatomically in the context of pancreatic cancer. For lymph nodes around the superior mesenteric artery (group 14), the metastatic rate of the subgroup 14c (lymph nodes at the root of the medial colic artery) was 6.6%, thus encompassing group 14c may not be necessary in CTV for radiation therapy. Likewise, lymph nodes around the common hepatic artery (group 8) have a number of subgroups. The metastatic rate to group 8p (lymph nodes of the posterior region) was seen in 6.8% of cases. However, since such finding was seen in only one study, exclusion of group 8p cannot be recommended.

The collective evidence also demonstrated that the probability of metastasis to nodal group 1-7 described by the JPS including peri-gastric and infrapyloric nodes are relatively rare (all less than 10%). Therefore, group 1-7 can be excluded from the high-dose coverage during precision radiation therapy. The metastatic rate to the hepatoduodenal ligament (group 12) was 7.9%, and could be considered as a "low-risk" region. However, once lymphatic invasion occurs, the rate of involvement raised to 20.7%. In addition, hepatoduodenal ligament group is a potential lymphatic route to hepatic metastasis [16]. Therefore, it is reasonable to encompass hepatoduodenal ligament group in the CTV unless lymphatic invasion is absent in pathology examination after complete resection.

The extent of cancer including that of lymph node metastasis is usually associated to certain characteristics of the disease such as the extent of the primary disease, differentiation, and lymphatic vessel invasion, etc. However, the collective data and analyses in the current study failed to demonstrate such correlation. As most of the patients included in the 18 studies in our analyses were surgically resectable, such counterintuitive finding could only demonstrated that lymph node metastasis may occur in the earliest stage of pancreatic cancer. Such phenomenon may indicate the important of adjuvant therapy in definitive treatment of pancreatic cancer, and reduced target volume may not be ideal even for small and/or well-differentiated tumors at early stages.

The most commonly involved lymph node regions in pancreatic body/tail cancers include those around the common hepatic artery, lymph nodes around the celiac trunk, lymph nodes along the splenic artery, lymph nodes around the superior mesenteric artery, paraaortic lymph nodes and inferior body lymph nodes. Therefore, these regions should be included in the target volumes. With limited data on pancreatic body/tail cancers, no correlation between lymph node metastatic rates and tumor characteristics was observed. Therefore, no change in CTV selection and delineation is recommended according to tumor characteristics for the pancreatic body/tail cancers.

Para-aortic lymph nodes, despite its more distant location in pancreatic cancer, have relatively high probability of disease involvement, according to 14 of the 18 studies included, for both head and body/tail cancers of the pancreas [1,16-20,23-28,30,31]. Para-aortic nodes can be categorized according to their anatomic position and the probability of metastasis in pancreatic cancer. Para-aortic lymph nodes anterior to the aorta and in-between aorta and vena cava from the celiac artery to the inferior mesenteric artery had much higher metastatic rates than those lateral and posterior to the aorta and vena cava and those anterior to the vena cava (metastatic rates all <4%). Therefore, high radiation dose regions should encompass at least the nodes anterior and medial to the aorta between celiac and inferior mesenteric artery.

One of the recent developments in image technology that may provide more accurate selection and delineation of CTV in pancreatic cancer is the use of functional image studies. The prevailing use of functional imaging especially FDG-PET/CT may provide more accurate diagnosis of regional node diagnosis in many neoplasms. And the effectiveness of FDG-PET/CT in the selection and delineation of both primary tumor and regional metastasis has been demonstrated in a number of malignancies including lung cancer and head and neck cancers [39-41]. The sensitivity and specificity of FDG-PET/CT in the diagnosis and evaluation of pancreatic cancer were reportedly 71-100% and 64-95%, respectively, significantly higher than those of CT scans [42,43]. However, false-positive FDG-PET findings may be seen in inflammatory conditions, while hyperglycemia and small tumor sizes may results in false-negative results. In addition, most of the lymph node metastasis remains undetectable because of their small size, for which a low sensitivity range between 20%-35% was observed [36,44]. Clearly, the capability of FDG-PET/CT in detecting subclinical disease in lymph node is limited, and the use of the results of FDG-PET/CT to guide CTV-node delineation is not feasible at this time.

The pathologic findings summarized in the current analyses represent a factorial summary of the pattern of lymph node metastasis in patients with resectable pancreatic cancer. However, the clinical value, i.e., the application of such results in clinical practice is largely unknown. In resectable pancreatic cancer, the extent of treatment to the lymph node is controversial. The results of a number of retrospective studies from Japan indicated that extended lymphadenectomy were associated with improved long-term survival, and the 5-year overall survival of patients underwent extended lymphadenectomy approached 30%-35% [15,45]. However, such findings were not universally supported from the results of prospective randomized clinical trials published recently. Results from most trials indicated that although extended lymphadenectomy showed similar perioperative morbidity and mortality as standard lymphadenectomy, no long-term survival benefits were identified [46-48]. Two of these studies reported severe diarrhea in a high percentage of patients after extended lymphadenectomy [47,48]. Although most physicians would agree on the necessity to resect regional lymphatic areas with high metastatic rate electively [3,17,18,20,49], the efficacy of radiation therapy to those areas, with or without extended lymphadenectomy, remains a focus of clinical trial. In addition, more than 85% of pancreatic cancer cases present as locally advanced and unresectable disease, and regional metastasis to lymph nodes may be more extensive than in resectable cases.

Under these circumstances, the collective data presented in the current analyses serves as a hypothesis generated for further clinical trials, preferably in prospective fashion, to investigate the efficacy of elective radiation therapy to the regional lymph nodes in adjuvant or definitive treatment using precision radiation therapy. However, such effort is further complicated by reduced accuracy of target volume contouring due to movement caused by respiration [50], and increased treatment volume to compensate such inaccuracy may cause intolerable toxicity if high-dose radiation therapy is implemented [51,52]. In addition, isolated recurrence in the regional lymph nodes is rare in pancreatic cancer, and the main mode of treatment failure include both local/regional and distant metastasis. Nevertheless, improvement in outcome including systemic disease control has been observed with the use of more effective chemotherapy agent such as gemcitabine [53]. Furthermore, the concurrent use of chemotherapy and IMRT in definitive treatment of pancreatic has been reported [54,55]. To further evaluate the efficacy of concurrent gemcitabine and IMRT with dose escalation under active breathing control (ABC) in the treatment of unresected locally advanced pancreatic cancer, a Phase II clinical trial has been initiated in the participating institutions of the current analyses. However, due to the limited number of patients who present with early stage pancreatic cancer and could achieve complete resection, investigation on the clinical value of adjuvant radiation therapy to the high-risk nodal regions in resectable pancreatic cancer will not be possible without multi-institutional effort.

## Conclusions

Regional lymph node metastases in pancreatic cancer follow a predictable pattern based on the origin of the disease within pancreas. Clinical target volume of radiation therapy for subclinical disease should be designed with consideration of the probability of nodal metastasis. Although clinical investigation is needed to validate the efficacy of elective radiation therapy to the high-risk regions, the suggested strategy based on pooled analyses of clinical evidences forms a reasonable recommendation of CTV-Node definition in precision radiation therapy of resectable pancreatic cancer.

## Competing interests

The authors declare that they have no competing interests.

## Authors' contributions

WJS collected and analyzed data and performed statistical analysis. WJS and JL drafted the manuscript. CNL reviewed the data and revised the manuscript. ZZ and JL designed the study and revised the final version. All authors have read and approved the final version of the manuscript.
